# Response to Letter to the Editor From Tapia-Castillo et al: Considerations About the Indirect Role of Low Cortisone in Subjects With Normal Cortisol to Cortisone Ratio

**DOI:** 10.1210/jendso/bvae139

**Published:** 2024-07-27

**Authors:** Decio Armanini, Chiara Sabbadin

**Affiliations:** Department of Medicine (DIMED), University of Padua, 35128 Padua, Italy; Unit of Endocrinology, University Hospital of Padua, 35128 Padua, Italy; Department of Medicine (DIMED), University of Padua, 35128 Padua, Italy; Unit of Endocrinology, University Hospital of Padua, 35128 Padua, Italy

**Keywords:** low-renin hypertension, obesity, inflammation, aging, cortisol, cortisone, 11β- hydroxysteroid dehydrogenase

We appreciated the letter of Tapia-Castillo et al [1] regarding our commentary on their paper [2]. In support of the interpretation of their results, the Authors refer their previous publication [3] where they evaluated the expression of 11β-hydroxysteroid dehydrogenase type 1 (11βHSD1) in liver and visceral adipose tissue in extremely obese subjects and concluded that in liver there is an overexpression of 11βHSD1, but the peripheral cortisol (F) levels remain normal because of hepatic metabolism of F and cortisone (E) by 5α- and 5β-reductases. It is well-known that circulating free F and E are regulated by adrenocorticotropin (ACTH), while the F to E ratio (F/E) is mainly dependent on the activity of 11βHSD. In their publication, the authors did not find [4] an increase of serum F, only a decrease of E and renin, and this discrepancy highlights the complexity of the regulation of F and E. 11βHSD1 is expressed in many tissues, playing a physiological role in healthy people and a pathological one in many diseases, particularly in inflammatory situations, such as obesity, hypertension, and aging, three clinical situations where E and renin were lower [2].

Regarding our publication on PAI-1 [5], in this study we evaluated the inflammatory effect of high amounts of aldosterone in vitro in mononuclear leukocytes (MNLs) of healthy subjects. We previously reported that MNL possess mineralocorticoid receptors (MRs), glucocorticoid receptors, 11βHSD1 but not 11βHSD2 [6], supporting the hypothesis that F could play a role in regulating the mineralocorticoid effector mechanism in the site of inflammatory reaction.

An important factor to be considered is the availability of NAD or NADP in the circulating and infiltrating MNLs. NAD is necessary for the correct function of 11βHSD2 and NADP for 11βHSD1. In inflammatory diseases the reduced NADP availability can affect 11βHSD1 function independently from its expression, since NADP is consumed for the inflammatory reaction in infiltrating MNLs, macrophages, and dendritic cells. The consequent decreased concentration of F in these cells can allow the binding of aldosterone to MR amplifying the inflammatory response. This situation is more evident in kidney, which presents MRs, glucocorticoid receptors, 11βHSD1, and 11βHSD2. In inflammatory conditions, the reduced availability of NAD in cells containing 11βHSD2 or of NADP in those containing 11βHSD1 could be involved in the final concentrations of aldosterone, renin and E.

ACTH can also be involved in the mineralocorticoid effector mechanism: (1) ACTH stimulates the secretion of aldosterone in situations of stress, (2) F can directly activate the renin–angiotensin–aldosterone system, and (3) renin decreases with aging.

In conclusion, the importance of the study of Tapia-Castillo et al [4] is the finding of low E in low renin hypertension, obesity, and aging, but the explanation of this finding cannot be related only to 11βHSD2, which is certainly the main regulator of the F/E. The definition of hormones is related to the fact that they circulate, and the values of F and E are directly or indirectly regulated by the balance of all the factors reported in our commentary.

## Disclosures

The authors have nothing to disclose. Decio Armanini is an Editorial Board Member for Journal of the Endocrine Society and played no role in the Journal's evaluation of the manuscript.

## Abbreviations

11βHSD1: 11β-hydroxysteroid dehydrogenase type 1
ACTH: adrenocorticotropin
E: cortisone
F: cortisol
MNL: mononuclear leukocyte
MR: mineralocorticoid receptor
